# The effect of homocysteine-lowering with B-vitamins on osteoporotic fractures in patients with cerebrovascular disease: substudy of VITATOPS, a randomised placebo-controlled trial

**DOI:** 10.1186/1471-2318-13-88

**Published:** 2013-09-03

**Authors:** John Gommans, Qilong Yi, John W Eikelboom, Graeme J Hankey, Christopher Chen, Helen Rodgers

**Affiliations:** 1 Department of Medicine, Hawke’s Bay Hospital, Hastings, New Zealand; 2 National Epidemiology and Surveillance, Canadian Blood Services, Ottawa, Canada; 3 Department of Medicine, McMaster University, Hamilton, Ontario, Canada; 4 School of Medicine and Pharmacology, The University of Western Australia, Nedlands, Perth, Australia; 5 Department of Pharmacology, National University of Singapore, Singapore, Republic of Singapore; 6 Stroke Research Group, Institute for Ageing and Health, Newcastle University, Newcastle upon Tyne, England, UK

**Keywords:** Homocysteine, B-vitamins, Osteoporosis, Fractures, Stroke

## Abstract

**Background:**

Homocysteine has been postulated as a novel, potentially reversible risk factor for osteoporosis and related fractures. We evaluated whether homocysteine-lowering therapy with B-vitamins in patients with symptomatic cerebrovascular disease reduced the incidence of osteoporotic fractures.

**Methods:**

VITAmins To Prevent Stroke (VITATOPS) was a prospective randomised double-blind placebo-controlled trial in which 8,164 patients with recent (within 7 months) stroke or transient ischemic attack were randomly allocated to double-blind treatment with one tablet daily of either placebo (n = 4,075) or B-vitamins (folic acid 2 mg, vitamin B_6_ 25 mg, vitamin B_12_ 500 μg; n = 4,089). Patients were reviewed every six months. Any osteoporotic fracture and osteoporotic hip fractures were secondary outcome events, and were reviewed by a masked adjudication committee. Analysis was by intention to treat. Logistic regression was used to identify independent predictors of fracture.

**Results:**

Participants had a mean age of 62.6 years (SD 12.5 years) and 64% were male, 42% of Western European descent and 75% were functionally independent (Oxford Handicap Scale of two or less). After a median duration of 2.8 years therapy and 3.4 years follow-up, there was no significant difference in the incidence of any osteoporotic fracture between participants assigned B-vitamins (67 [1.64%]) and placebo (78 [1.91%]; risk ratio [RR] 0.86, 95% confidence interval [CI] 0.62-1.18) or the incidence of hip fractures (34 [0.83%] B-vitamins vs. 36 [0.88%] placebo; RR 0.94, 95% CI 0.59-1.5). There was no significant impact of B-vitamin therapy on time to first fracture. Baseline homocysteine levels did not predict any osteoporotic fracture (p =0.43). Independent predictors of any osteoporotic fracture were female sex, age > 64 years, Western European ethnicity and use of anti-osteoporosis medication at randomization (all p < 0.01).

**Conclusions:**

Once daily treatment with B-vitamins had no effect on incidence of osteoporotic fractures during a median of 3.4 years follow-up in patients with cerebrovascular disease. A modest effect of B-vitamin therapy is not excluded due to the low numbers of fracture outcome events.

**Trial registration:**

Clinicaltrials.gov number: NCT00097669 and isrctn.org number: ISRCTN74743444.

## Background

Osteoporosis is a widely prevalent, age-related skeletal disorder characterized by compromised bone strength and an increased risk of bone fracture [1,2]. Osteoporotic fractures, particularly hip (femoral neck) fractures that usually require hospitalisation and surgery, are frequently associated with poor outcomes including the need for institutional care and death [1,3]. Simple interventions that can prevent or delay the onset of osteoporosis and its complications are needed to stem the projected increase in the burden of osteoporosis due to the increasing age and other demographic changes in the population [4,5].

An increased plasma concentration of total homocysteine (tHcy) has been postulated as a novel, potentially treatable risk factor for osteoporotic fractures, including hip fractures [2,6-8]. In one prospective population-based study, the highest quartile of tHcy was associated with a two-fold increase in risk of fracture (risk ratio [RR] 1.9, 95% confidence interval [CI] 1.4-2.6), and the association was continuous; with each standard deviation (SD) increase in tHcy, the risk of fracture increased by 30% (RR 1.3, 95% CI 1.1-1.5) [9]. These findings have been validated in other populations [10,11]. Further, the strength of the association between high tHcy and fracture risk (RR 1.9 for every SD increase) is of similar magnitude to that attributable to other accepted risk factors for hip fracture such as age over 75 years (RR 2.3), falls in the previous year (1.9), low bone density (1.6) and current smoking (1.6) [9]. Several biological mechanisms have been postulated to account for this association but it is still uncertain whether the association is causal, confounded or biased due to reverse causality [2,6,12-14]. As raised tHcy can be lowered with B-vitamin therapy, [15,16] it is possible to determine whether tHcy is a causal risk factor for bone fracture by means of randomised controlled trials (RCTs) of B-vitamins vs. placebo. Four RCTs have examined the effect of B-vitamins on biomarkers of bone turnover but have been limited by the use of surrogate outcomes for fracture risk, lack of statistical power due to the inclusion of small numbers of subjects and/or use of healthy volunteers [17-20]. Two RCTs of B-vitamins on bone fracture risk have produced conflicting results. The largest, involving 5522 patients, failed to show any significant impact of B-vitamins on fracture incidence despite a 3.3 μmol/L lower tHcy and a mean follow-up of five years [21,22]. The only positive intervention study to date was undertaken in 628 elderly Japanese patients with significant residual hemiplegia at least one year following stroke [23]. Those treated with combination folate and vitamin B12 had a 75% reduction (adjusted RR 0.24, 95% CI 0.11-0.53) in the incidence of fracture at two years. However, the study population was highly selected and characterized by severe disability, very high homocysteine concentrations (mean 19.9 μmol/L), and an unusually high fracture rate in the control group, approximately 10 times the rate found in the average Japanese population of the same age [24].

In light of the uncertainty surrounding the role of tHcy in the pathogenesis of osteoporosis and bone fracture, we prospectively examined the occurrence of any osteoporotic fracture and osteoporotic hip fractures in the VITAmins TO Prevent Stroke (VITATOPS) trial of patients with recently symptomatic cerebrovascular disease treated with either B-vitamins or placebo.

## Methods

The rationale, methods, CONSORT flowchart and primary results of the VITATOPS trial have been published [25,26]. In summary, VITATOPS was an investigator-led, prospective, randomised, double-blind, placebo-controlled trial in which 8164 patients with recent (within seven months) stroke or transient ischemic attack (TIA) were recruited between November 19 1998 and December 31 2008, and randomly assigned to receive either one tablet daily of B-vitamins (folic acid 2 mg, vitamin B_6_ 25 mg, vitamin B_12_ 500 μg) or matching placebo. Final follow-up was 30 June 2009 ensuring a minimum six months and up to 10.5 years on study medication.

The study was conducted in accordance with principles of good clinical practice and the Declaration of Helsinki, and received ethics approval in the United Kingdom from the Multicentre Research Ethics Committee for Scotland A, in New Zealand from the Multi-region Ethics Committee and from local research ethics committees applicable to each participating centre. A full list of the 127 participating centres is available in an additional file [see Additional file 1]. All patients provided written informed consent before enrolment.

Demographic and clinical characteristics of the participants recorded at baseline included; age, sex, ethnicity (of participants and their parents and grandparents), clinical details of the qualifying cerebrovascular event, current medications, past medical history (major vascular events, revascularisation procedures, depression), vascular risk factors (hypertension, smoking, hypercholesterolaemia, diabetes mellitus, ischaemic heart disease, atrial fibrillation, peripheral artery disease), alcohol intake, Oxford Handicap Score, Hospital Anxiety and Depression Score, and Mini Mental State Examination score. Cox’s proportional hazards models were used to adjust for differences in baseline variables.

Investigators were encouraged but not obligated to take blood samples from study participants who consented to these optional tests, to measure blood concentrations of tHcy, red cell folate and vitamin B12 at study entry and/or at the time of their final follow-up visit. Fasting plasma tHcy was measured by high-performance liquid chromatography, on venous blood samples collected after an overnight fast.

The primary outcome measure for the main VITATOPS study was the composite of any stroke, myocardial infarction or death due to vascular causes. In this substudy the main outcome measure was occurrence of any clinically apparent osteoporotic fracture. Osteoporotic hip fractures and time till first fracture were secondary outcome measures. Non-osteoporotic fractures were excluded from analysis.

Patients were reviewed six-monthly and data on clinical fracture outcomes were based on information obtained from clinical history, radiology reports, hospital discharge reports and health records. All hospitalisations including those for fracture were recorded prospectively, and from 2004 (following publication of the potential association between homocysteine and fracture risk [6]) until the end of the trial in June 2009 all clinically apparent fractures whether hospitalised or not, were recorded prospectively.

A masked adjudication committee audited all fractures. Fractures were classified as osteoporotic if they were reported to be osteoporotic by the investigator; and involved the hip (neck of femur), wrist (distal radius) or spine (vertebral), or occurred spontaneously or in the context of minor trauma; and if there was no other apparent cause (e.g. severe trauma, metastatic cancer).

The primary statistical analysis for this substudy was an intention-to-treat analysis of the incidence of any osteoporotic fracture in the placebo and B-vitamin treatment groups over the duration of follow-up. This was added to the pre-established analysis plan in 2004. A secondary per protocol on-treatment analysis would exclude any patients found to be invalid after randomisation or who had cross-over in treatment during follow-up.

The event rates were calculated as the number of events that occurred during the follow-up period divided by the total number of patients randomised. The risk ratios (treatment/placebo) of the fracture rates in the treatment and placebo groups and their 95% confidence intervals were calculated to describe the treatment effect. We used Kaplan-Meier methods to construct cumulative time-to-event curves for the two groups and the main comparison was based on a log-rank test.

We did stepwise logistic multivariable regression analysis to determine the independent risk factors for osteoporotic fracture in the study population.

Two sided significance tests were used throughout and a two-sided p-value < 0.05 was considered significant.

## Results

Full details of baseline demographic data, clinical characteristics, laboratory results, adherence to therapy, follow up rates and primary (non-fracture) outcome data have previously been published [26]. Participants had a mean age of 62.6 years (SD 12.5 years) and 64% were male, 42% of Western European descent and 75% had an Oxford Handicap Scale of two or less indicating either no lifestyle restrictions or ability to manage without assistance.

At randomisation, 4089 participants were allocated to B-vitamins and 4075 to placebo. A total of 579 participants (7.1% B-vitamins, 6.8% placebo, p = 0.39) were taking medications for prevention or treatment of osteoporosis. These were predominantly calcium supplements - alone (n = 462) or in combination with other medications (n = 41). The remainder were vitamin D (n = 24), bisphosphonates (n = 24), hormone replacement therapy (n = 18) or a combination of these (n = 10).

### Follow-up

Follow-up continued for a median duration of 3.4 years per person (Inter Quartile Range [IQR] 2.0-5.5 years, range 0–9 years) with mean adherence to study treatment of 2.8 years (IQR 1.2-4.7 years). The longer duration of follow-up (0.6 years) reflects those patients who discontinued trial drugs but agreed to continue with follow-up.

A total of 425 patients (5.2%) withdrew from study follow-up prior to the inclusion of fractures as a specified reportable outcome event in 2004.

### Osteoporotic fracture incidence

**Figure 1:**
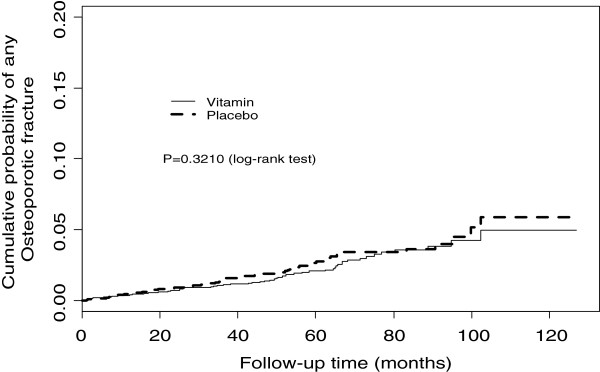
Kaplan Meier estimates of any osteoporotic fracture outcomes.

**Figure 2:**
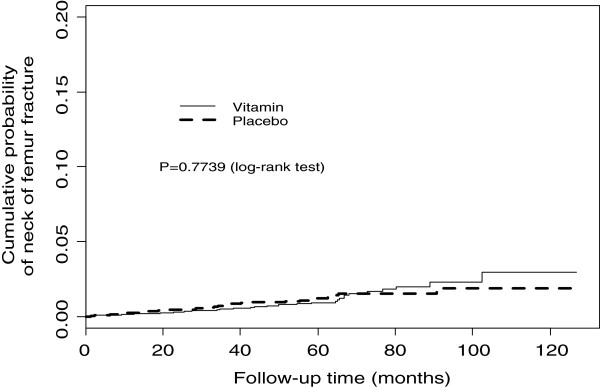
Kaplan Meier estimates of osteoporotic hip fracture outcomes.

At the end of the trial, any clinically apparent osteoporotic fracture occurred in 67 (1.64%) participants assigned B-vitamins and 78 (1.91%) assigned to placebo (RR 0.86, 95% CI 0.62-1.18); and hip fractures occurred in 34 (0.83%) assigned B-vitamins and 36 (0.88%) assigned to placebo (RR 0.94, 95% CI 0.59-1.5) (Table 1). There was no significant impact of treatment on any other osteoporotic fracture subtype. A subgroup analysis excluding the 425 (5.2%) patients who left the study prior to the 2004 inclusion of all fractures as a specified reportable outcome event, rather than just hospitalised fractures, did not alter the findings. Compared with placebo, treatment with B-vitamins was not associated with any significant difference in the time to first fracture for any osteoporotic fracture or hip fractures (Figures 1 and 2). A secondary per protocol on-treatment analysis also showed no impact of treatment on any osteoporotic fracture outcome after removal of patients found to be invalid after randomisation or who had cross-over in treatment during follow-up; either placebo group patients taking B-vitamins or B-vitamin group patients stopping their treatment (p = 0.85).

**Table 1:** Osteoporotic fracture outcomes according to treatment group

**Osteoporotic fractures**	**B-Vitamins**	**Placebo**	**Relative risk**	**p-value**
	**(n = 4089)**	**(n = 4075)**	**(95% confidence interval)**	**(log-rank test)**
	**N (%)**	**N (%)**		
Any	67 (1.6)	78 (1.1)	0.86 (0.62,1.18)	0.32
Hip	34 (0.8)	36 (0.9)	0.94 (0.59,1.50)	0.77
Wrist	7 (0.2)	8 (0.1)	0.87 (0.32,2.40)	0.78
Thoracic spine	4 (0.1)	5 (0.1)	0.80 (0.21,2.97)	0.70
Other	26 (0.6)	35 (0.9)	0.74 (0.45,1.23)	0.24

Note: Only first fracture was used for each type of events. One patient could have multiple different types of fractures, so sum of fracture types could be more than the total of fractures.

### Predictors of osteoporotic fracture

On multivariate analysis independent predictors of any osteoporotic fracture were female sex (adjusted odds ratio [OR] 2.58, 95% CI 1.80-3.70), age > 64 years (OR 4.18, 95% CI 2.60-6.72), Western European ethnicity (OR 3.86, 95% CI 2.52-5.92) and use of anti-osteoporosis medication at randomization (OR 1.78, 95% CI 1.11-2.86). (Tables 2 and 3). In participants who volunteered to have their homocysteine levels measured at baseline (n = 1205), there was no difference between those with (n = 40; mean tHcy 14.2 ±6.22 μmol/L) or without subsequent fracture (n = 1165; mean tHcy 14.3 ±8.56 μmol/L, p = 0.43). Homocysteine levels at follow-up were not associated with fracture outcomes in either treatment group (Table 4).

**Table 2:** Baseline characteristics for patients with and without any osteoporotic fracture

**Baseline characteristic**		**With any fracture**	**Without fracture**	**p-value**
		**(n = 145)**	**(n = 8019)**	**(log-rank test)**
		**N (%)**	**N (%)**	
Sex	Male	55 (37.9)	5163 (64.4)	<.001
	Female	90 (62.1)	2854 (35.6	
Age	<65 yrs	23 (15.9)	4276 (53.3)	<.001
	> = 65 yrs	122 (84.1)	3743 (46.7)	
Ethnicity	Western European	105 (78.9)	3171 (42.3)	<.001
	Oriental	15 (11.3)	1898 (24.7)	
	South Asian	7 (5.3)	2046 (26.6)	
	Other	6 (4.5)	566 (7.4)	
Oxford Handicap Score	> = 3	35 (24.7)	1859 (23.8)	0.18
	<3	107 (75.4)	5952 (76.2)	
Smoking (ever)	Yes	72 (49.7)	3947 (49.9)	0.43
	No	73 (50.3)	3966 (50.1)	
Alcohol (any)	Yes	54 (37.2)	2269 (28.3)	0.20
	No	91 (62.8)	5748 (71.7)	
Diabetes	Yes	24 (16.6)	1875 (23.7)	0.21
	No	121 (83.5)	6047 (76.3)	
Stroke	Yes	28 (19.3)	1254 (15.8)	0.28
	No	117 (80.7)	6664 (84.2)	
Myocardial infarction	Yes	15 (10.3)	583 (7.4)	0.33
	No	130 (89.7)	7317 (92.6)	
Hypertension	Yes	105 (72.4)	5632 (71.1)	0.45
	No	40 (27.6)	2285 (28.9)	
Depression	Yes	16 (16.0)	528 (7.4)	0.003
	No	84 (84.0)	6650 (92.6)	
Antidepressant medication	Yes	24 (16.6)	536 (6.8)	<.001
	No	121 (83.5)	7332 (93.2)	
Corticosteroid medication	Yes	3 (2.1)	47 (0.6)	0.011
	No	142 (97.9)	7972 (99.4)	
Any Osteoporosis Medication *	Yes	23 (15.9)	556 (6.9)	<.001
	No	122 (84.1)	7463 (93.1)	
Homocysteine level at baseline †		(n = 40)	(n = 1165)	0.43
mean (SD)		14.2 (6.2)	14.3 (8.6)	

* Including Calcium, Vitamin D, Bisphosphonates and Hormone replacement therapy;

† Not all participants had homocysteine levels measured at Baseline.

**Table 3:** Any osteoporotic fracture outcomes according to baseline characteristics and treatment group

**Baseline characteristic**		**B-Vitamins**	**Placebo**	**p-value**	**P-value for interaction**
		**(n = 4089)**	**(n = 4075)**	**(log-rank test)**	
		**N (%)**	**N (%)**		
Sex	Male	27 (1.0)	28 (1.1)	0.90	0.50
	Female	40 (2.7)	50 (3.4)	0.21	
Age	<65 yrs	12 (0.6)	11 (0.5)	0.90	0.57
	> = 65 yrs	55 (2.8)	67 (3.5)	0.28	
Ethnicity	Western European	48 (2.9)	57(3.5)	0.35	0.65
	Oriental	9 (0.9)	6 (0.6)	0.42	
	South Asian	4 (0.4)	3 (0.3)	0.74	
	Other	1 (0.4)	5 (1.7)	0.12	
Oxford Handicap Score	> = 3	14 (1.5)	21 (2.2)	0.18	0.34
	<3	51 (1.7)	56 (1.9)	0.63	
Smoking (ever)	Yes	36 (1.8)	36 (1.8)	0.96	0.30
	No	31 (1.5)	42 (2.1)	0.16	
Alcohol (any)	Yes	26 (2.2)	28 (2.5)	0.71	0.76
	No	41 (1.4)	50 (1.7)	0.32	
Diabetes	Yes	9 (0.9)	15 (1.6)	0.21	0.34
	No	58 (1.9)	63 (2.0)	0.62	
Stroke	Yes	13 (2.1)	15 (2.3)	0.75	0.94
	No	54 (1.6)	63 (1.9)	0.38	
Myocardial infarction	Yes	8 (2.7)	7 (2.3)	0.71	0.49
	No	59 (1.6)	71 (1.9)	0.25	
Hypertension	Yes	48 (1.7)	57 (2.0)	0.40	0.98
	No	19 (1.6)	21 (1.8)	0.64	
Depression	Yes	8 (2.9)	8 (3.0)	0.83	0.51
	No	35 (1.0)	49 (1.5)	0.14	
Antidepressant medication	Yes	11 (4.0)	13 (4.6)	0.67	0.99
	No	56 (1.5)	65 (1.8)	0.37	
Corticosteroid medication	Yes	10 (3.3)	13 (4.7)	0.05	0.98
	No	57 (1.5)	65 (1.7)	0.46	
Osteoporosis Medication	Yes	4 (1.3)	5 (1.8)	0.37	0.62
	No	30 (0.8)	31 (0.8)	0.44	
Homocysteine	< median	10 (3.2)	10 (3.5)	0.77	0.81
(median 12.6 micromol/L)	(n = 595)				
	> = median	8 (2.7)	12 (3.8)	0.53	
	(n = 610)				

**Table 4:** Mean homocysteine concentration at follow up* according to treatment group and fracture outcomes

	**With fracture mean (standard deviation)**	**Without fracture mean (standard deviation)**	**Difference, (95% confidence interval)**	**P-value (paired t-test)**
Homocysteine (micromol/L				
Placebo (n = 584)	15.7 (5.9) (n = 23)	14.2 (6.1) (n = 561)	−1.52 (−4.08,1.04)	0.2447
B-Vitamins (n =580)	11.7 (9.6) (n = 18)	10.5 (4.6) (n = 562)	−1.17 (−3.46,1.11)	0.3118

* The difference between baseline and follow-up Homocysteine levels were also compared between patients with and without facture. The differences are not significant, either (data not shown).

Note: Only a small subset of patients (14.3%) had homocysteine levels measured at final follow up.

### Effect of trial medication on homocysteine, folate and vitamin B12 levels

Of the 1164 (14.3%) study participants who volunteered to have optional fasting plasma tHcy levels measured at their final follow-up visit, patients in the B-vitamins group had a mean 3.8 μmol/L (95% CI 3.1-4.4 μmol/L) lower tHcy level compared with placebo (10.5 vs. 14.3 μmol/L, p < 0.001). Table 5 compares folate and vitamin B12 levels between baseline and final follow up visits in the small subset that volunteered to provide these.

**Table 5:** Mean homocysteine, red cell folate and vitamin B12 concentration at baseline and follow up according to treatment group

	**Baseline: mean (standard deviation)**	**Follow-up: mean (standard deviation)**	**Difference (95% confidence interval)**	**P-value (paired t-test)**
**Homocysteine** (micromol/L)				
Placebo (n = 463)	13.4 (5.0)	14.3 (5.7)	0.88 (0.40,1.36)	<0.001
B-Vitamins (n = 462)	13.5 (6.3)	10.5 (4.2)	−3.06 (−3.53,-2.59)	<0.001
**Red cell Folate (**nmol/L)				
Placebo (n = 173)	895.0 (466.6)	1135.2 (666.8)	244.8 (151.0,338.7)	<0.001
B-Vitamins (n = 183)	959.2 (464.1)	2181.5 (752.3)	1220.0 (1117,1341)	<0.001
**B 12** (pmol/L)				
Placebo (n = 190)	317.6 (140.8)	315.3 (188.9)	−2.3 (−26.4,31.1)	0.87
B-Vitamins (n – 196)	322.1 (150.8)	594.7 (271.9)	272.6 (235.2,310.0)	<0.001

Note: Only small subsets of patients had homocysteine, folate and vitamin B12 measured at both baseline and follow up.

### Adverse events

Vitamin B12 deficiency was diagnosed during follow up in none of the 4089 patients in the B vitamins group compared with six (0.1%) of 4075 patients in the placebo group (p = 0.02). Peripheral neuropathy suspected to be caused by vitamin B6 toxicity was diagnosed in five patients assigned to B vitamins (0.1%) compared with nine patients assigned to placebo (0.2%; p = 0.30). There were no unexpected serious and non-serious adverse events and there were no significant differences in common adverse effects between the treatment groups (data not shown).

## Discussion

This study did not identify any effect of daily treatment with B-vitamins for a median of 2.8 years on the overall incidence of any clinically apparent osteoporotic fracture or osteoporotic hip fractures during a median of 3.4 years follow-up in 8,164 patients with recent stroke or TIA participating in the VITATOPS study.

The study findings of a lack of effect of B vitamins on osteoporotic fracture are consistent with those of the HOPE-2 trial, in which 5522 patients at risk of vascular disease were treated with B-vitamin therapy for a prolonged period (mean 5 years) and tHcy was lowered by a similar amount (3.8 μmol/L in VITATOPS and 3.3 μmol/L in HOPE-2) compared with placebo [20,21]. Our results in patients with recent stroke or TIA are at odds however with the positive findings of the smaller trial of B-vitamins in disabled Japanese stroke survivors with high tHcy concentrations [23].

The study’s strengths include the large number of patients (8164) and their recruitment from a broad range of centres, countries and ethnic groups; the randomization process minimising any systematic bias in treatment allocation; and the blinding of all assessors, clinicians and patients to treatment allocations minimising observer bias.

The most significant limitation of the study was that it was underpowered to reliably identify or exclude a modest but still clinically important effect of B-vitamins on fracture rates as there was only a small number of fracture events resulting in wide 95% confidence intervals for the estimate of relative risk for any fracture with B-vitamin therapy compared with placebo - ranging from up to a 38% reduction to an 18% increase. The low absolute fracture rate most likely reflects the study population with predominance of men (64%), people of non-Western European descent (58%) and younger patients (mean age 62.6 years) who are recognised to have lower rates of fracture [1,27]. Further, most patients with stroke or TIA enrolled in the trial were functionally independent (75%) and therefore not at high risk of falls and fractures [28] unlike those recruited into the positive study of Japanese stroke survivors [23]. In addition, few patients had pre-existing osteoporosis at time of randomisation, as identified by low concurrent use of common osteoporosis preventative and treatment medications (7%). To reliably detect a modest but potentially clinically important 15% relative risk reduction (RRR) with 80% power in this study population who appear to be at relatively low risk of fracture would require a study with over 75,000 participants; more than nine times that recruited to VITATOPS. Even if a statistically significant 14% RRR were proven, an absolute risk reduction of 0.27% (B-vitamins 1.64% and placebo 1.91%) would equate to a number needed to treat of 370 to prevent one fracture in 3.4 years.

Adherence to study medication was also incomplete, although similar between the two treatment groups. This may have biased the results toward the null hypothesis and reflects the difficulties inherent in investigator-led trials conducted with limited peer-reviewed funding [29].

It is possible that the median duration of adherence to treatment (2.8 years) and follow up (3.4 years) in VITATOPS was insufficient to identify or exclude any longer-term effects of therapy with B-vitamins on fracture risk. However, this is longer than most other published studies, with the sole exception of the five-year follow-up in the HOPE-2 trial [17-23].

An additional limitation was that ascertainment of all fracture outcome events may have been inadequate due to incomplete identification of minor fractures as efforts were focussed on identifying major vascular events, the primary study outcome. The relatively high proportion of all fracture outcomes due to hip fractures (48%) supports this possibility. Fracture ascertainment may also have been incomplete prior to prospective reporting of fractures in 2004; however, exclusion of those patients randomised before 2004 did not alter the findings. The study results were consistent when analysis was restricted to participants with hip fractures, an event that usually results in hospitalisation and is readily identified.

## Conclusions

In summary this study did not identify any effect of once daily therapy with folic acid, vitamin B_6_ and vitamin B_12_ for a median of 2.8 years on the overall incidence of osteoporotic fractures during a median of 3.4 years follow-up in 8,164 patients with recent stroke or TIA participating in the VITATOPS study.

A modest effect of B-vitamin therapy is not excluded due to the low numbers of fracture outcome events. Whilst awaiting the results of further prospective randomized controlled studies [30] our data suggests no adverse or favourable effect of homocysteine-lowering with B-vitamins on fracture outcomes.

## Competing interests

The authors declare that they have no competing interests.

## Authors’ contributions

GJH (Principal Investigator and Study Chair) and JWE designed the VITATOPS study. All authors were members of the VITATOPS International Steering Committee (JG, JWE, GJH and CC) or Data Monitoring Committee (QY) and/or investigators in the VITATOPS trial (JG, GJH, CC and HR). JG initiated the analysis for this substudy and wrote the first draft of the manuscript. All authors contributed to revision of manuscript content and approved the final version of the manuscript. QY takes responsibility for the integrity of data analysis.

## Pre-publication history

The pre-publication history for this paper can be accessed here:

http://www.biomedcentral.com/1471-2318/13/88/prepub

## Supplementary Material

Additional file 1 **Title of data is “VITATOPS trial: study group members, and investigators and centres”.** Data lists the members of the various VITATOPS trial study groups and the participating trial investigators and centres with their recruitment numbers. Click here for file

## References

[B1] NIH Consensus Statement Osteoporosis prevention, diagnosis, and therapy JAMA 2001 285 785 795 10.1001/jama.285.6.785 11525451

[B2] Herrmann M Schmidt JP Umanskaya N Wagner A Taban-Shomal O Widmann T Colaianni G Wildemann B Herrmann W The role of hyperhomocysteinemia as well as folate, vitamin B(6) and B(12) deficiencies in osteoporosis: a systematic review Clin Chem Lab Med 2007 45 1621 1632 18067447 10.1515/CCLM.2007.362

[B3] Melton LJ 3rd Adverse outcomes of osteoporotic fractures in the general population J Bone Miner Res 2003 18 1139 1141 10.1359/jbmr.2003.18.6.1139 12817771

[B4] Lippuner K Golder M Greiner R Epidemiology and direct medical costs of osteoporotic fractures in men and women in Switzerland Osteoporos Int 2005 16 Suppl 2 S8 S17 15378232 10.1007/s00198-004-1696-0

[B5] Burge R Dawson-Hughes B Solomon DH Wong JB King A Tosteson A Incidence and economic burden of osteoporosis-related fractures in the United States, 2005–2025 J Bone Miner Res 2007 22 465 475 10.1359/jbmr.061113 17144789

[B6] Raisz LG Homocysteine and osteoporotic fractures–culprit or bystander? N Engl J Med 2004 350 2089 2090 10.1056/NEJMe048030 15141048

[B7] Dhonukshe-Rutten RA Pluijm SM de Groot LC Lips P Smit JH van Staveren WA Homocysteine and vitamin B12 status relate to bone turnover markers, broadband ultrasound attenuation, and fractures in healthy elderly people J Bone Miner Res 2005 20 921 929 10.1359/JBMR.050202 15883631

[B8] Herrmann M Widmann T Herrmann W Homocysteine - a newly recognised risk factor for osteoporosis Clin Chem Lab Med 2005 43 1111 1117 16197307 10.1515/CCLM.2005.194

[B9] van Meurs JB Dhonukshe-Rutten RA Pluijm SM Pluijm SMF van der Klift M de Jonge R Lindemans J de Groot LCPGM Hofman A Witterman JCM van Leeuwen JPTM Breteler MMB Lips P Pols HAP Uitterlinden AG Homocysteine levels and the risk of osteoporotic fracture N Engl J Med 2004 350 2033 2041 10.1056/NEJMoa032546 15141041

[B10] McLean RR Jacques PF Selhub J Tucker KL Samelson EJ Broe KE Hannan MT Cupples LA Kiel DP Homocysteine as a predictive factor for hip fracture in older persons N Engl J Med 2004 350 2042 2049 10.1056/NEJMoa032739 15141042

[B11] Gjesdal CG Vollset SE Ueland PM Refsum H Meyer HE Tell GS Plasma homocysteine, folate, and vitamin B 12 and the risk of hip fracture: the hordaland homocysteine study J Bone Miner Res 2007 22 747 756 10.1359/jbmr.070210 17295607

[B12] Levasseur R Bone tissue and hyperhomocysteinemia Joint Bone Spine 2009 76 234 240 10.1016/j.jbspin.2008.11.002 19217816

[B13] McLean RR Jacques PF Selhub J Fredman L Tucker KL Samelson EJ Kiel DP Cupples LA Hannan MT Plasma B vitamins, homocysteine, and their relation with bone loss and hip fracture in elderly men and women J Clin Endocrinol Metab 2008 93 2206 2212 10.1210/jc.2007-2710 18364381 PMC2435634

[B14] Gerdhem P Ivaska KK Isaksson A Pettersson K Vaananen HK Obrant KJ Akesson K Associations between homocysteine, bone turnover, BMD, mortality, and fracture risk in elderly women J Bone Miner Res 2007 22 127 134 17032146 10.1359/jbmr.061003

[B15] Stanger O Herrmann W Pietrzik K Fowler B Geisel J Dierkes J Weger M DACH-LIGA homocystein (german, austrian and swiss homocysteine society): consensus paper on the rational clinical use of homocysteine, folic acid and B-vitamins in cardiovascular and thrombotic diseases: guidelines and recommendations Clin Chem Lab Med 2003 41 1392 1403 14656016 10.1515/CCLM.2003.214

[B16] Homocysteine Trialists Collaboration Dose-dependent effects of folic acid on blood concentrations of homocysteine: a meta-analysis of the randomized trials Am J Clin Nutr 2005 82 806 812 16210710 10.1093/ajcn/82.4.806

[B17] Green TJ McMahon JA Skeaff CM Williams SM Whiting SJ Lowering homocysteine with B vitamins has no effect on biomarkers of bone turnover in older persons: a 2-y randomized controlled trial Am J Clin Nutr 2007 85 460 464 17284744 10.1093/ajcn/85.2.460

[B18] Herrmann M Stanger O Paulweber B Hufnagl C Herrmann W Folate supplementation does not affect biochemical markers of bone turnover Clin Lab 2006 52 131 136 16584059

[B19] Herrmann M Umanskaya N Traber L Schmidt-Gayk H Menke W Lanzer G Lenhart M Schmidt JP Herrmann W The effect of B-vitamins on biochemical bone turnover markers and bone mineral density in osteoporotic patients: a 1-year double blind placebo controlled trial Clin Chem Lab Med 2007 45 1785 1792 18020969 10.1515/CCLM.2007.352

[B20] Keser I Ilich JZ Vrkic N Giljevic Z Colic Baric I Folic acid and vitamin B12 supplementation lowers plasma homocysteine but has no effect on serum bone turnover markers in elderly women: a randomized, double-blind, placebo-controlled trial Nutr Res 2013 33 211 219 10.1016/j.nutres.2013.01.002 23507227

[B21] Sawka AM Ray JG Yi Q Josse RG Lonn E Randomized clinical trial of homocysteine level lowering therapy and fractures Arch Intern Med 2007 167 2136 2139 10.1001/archinte.167.19.2136 17954810

[B22] Lonn E Yusuf S Arnold MJ Sheridan P Pogue J Micks M McQueen MJ Probstfield J Fodor G Held C Genest J Jr Homocysteine lowering with folic acid and B vitamins in vascular disease N Engl J Med 2006 354 1567 1577 16531613 10.1056/NEJMoa060900

[B23] Sato Y Honda Y Iwamoto J Kanoko T Satoh K Effect of folate and mecobalamin on hip fractures in patients with stroke: a randomized controlled trial JAMA 2005 293 1082 1088 10.1001/jama.293.9.1082 15741530

[B24] van Meurs JB Uitterlinden AG Homocysteine and fracture prevention JAMA 2005 293 1121 1122 10.1001/jama.293.9.1121 15741537

[B25] VITATOPS Trial Study Group The VITATOPS (Vitamins to Prevent Stroke) Trial: rationale and design of an international, large, simple, randomised trial of homocysteine-lowering multivitamin therapy in patients with recent transient ischaemic attack or stroke Cerebrovasc Dis 2002 13 120 126 11867886 10.1159/000047761

[B26] VITATOPS Trial Study Group B vitamins in patients with recent transient ischaemic attack or stroke in the VITAmins TO Prevent Stroke (VITATOPS) trial: a randomised, double-blind, parallel, placebo-controlled trial Lancet Neurol 2010 9 855 865 20688574 10.1016/S1474-4422(10)70187-3

[B27] Hippisley-Cox J Coupland C Predicting risk of osteoporotic fracture in men and women in England and Wales: prospective derivation and validation of QFractureScores BMJ 2009 339 b4229 10.1136/bmj.b4229 19926696 PMC2779855

[B28] Moayyeri A Alrawi YA Myint PK The complex mutual connection between stroke and bone health Arch Biochem Biophys 2010 503 153 159 10.1016/j.abb.2010.06.023 20599661

[B29] Sandercock P Vitamin B supplements for prevention of stroke Lancet Neurol 2010 9 842 843 10.1016/S1474-4422(10)70188-5 20688573

[B30] van Wijngaarden JP Dhonukshe-Rutten RA van Schoor NM van der Velde N Swart KM Enneman AW van Dijk SC Bouwer-Brolsma EM Zillikens MC van Meurs JB Brug J Uitterlinden AG Lips P de Groot LC Rationale and design of the B-PROOF study, a randomized controlled trial on the effect of supplemental intake of vitamin B12 and folic acid on fracture incidence BMC Geriatr 2011 11 80 10.1186/1471-2318-11-80 22136481 PMC3266639

