# Supplementary material for: The effect of homocysteine-lowering with B-vitamins on osteoporotic fractures in patients with cerebrovascular disease: substudy of VITATOPS, a randomised placebo-controlled trial
Source: BMC Geriatr. 2013 Sep 3;13:88. doi: 10.1186/1471-2318-13-88 (PMC3848681; doi:10.1186/1471-2318-13-88)
Supplement: Additional file 1 — Title of data is “VITATOPS trial: study group members, and investigators and centres”. Data lists the members of the various VITATOPS trial study groups and the participating trial investigators and centres with their recruitment numbers. [file 1471-2318-13-88-S1.docx]

**VITATOPS trial study group members and trial investigators**

**VITATOPS trial study group**

*Steering committee* G J Hankey (chair), J W Eikelboom, R I Baker, A Gelavis, S C Hickling, K Jamrozik, F M van Bockxmeer, S Vasikaran.

*International steering committee* G J Hankey (Australia, chair), A Algra (Netherlands), C Chen (Singapore), M C Wong (Singapore), R Cheung (Hong Kong Special Administrative Region, China), L Wong (Hong Kong Special Administrative Region, China), I Divjak (Serbia and Montenegro), J Ferro (Portugal), G de Freitas (Brazil), J Gommans (New Zealand), S Groppa (Moldova), M Hill (Canada), J D Spence (Canada), K R Lees (UK), L Lisheng (China), J Navarro (Philippines), U Ranawaka (Sri Lanka), S Ricci (Italy), R Schmidt (Austria), A Slivka (USA), K Tan (Malaysia), A Tsiskaridze (Georgia), W Uddin (Pakistan), G Vanhooren (Belgium), D Xavier (India).

*Data monitoring and safety committee* J Armitage (chair), M Hobbs, M Le, C Sudlow, K Wheatley, Q Yi.

*Outcome and adverse events* *adjudication committee* W Brown, M Bulder, J W Eikelboom, G J Hankey, W K Ho, K Jamrozik, CJM Klijn, E Koedam, P Langton, E Nijboer, P Tuch.

*Trial management committee* J Pizzi (1999–present), M Tang (2000–present), R Alaparthi (2009–present), M Antenucci (2006), Y Chew (2006–08), D Chinnery (2001–03), C Cockayne (2004–09), R Holt (August–October, 2009), K Loh (1999–2009), L McMullin (2003–04), G Mulholland (July, 2009–January, 2010), B Nahoo (July–October, 2009), E Read (August, 2009–November, 2009), F Smith (2002–09), C Y Yip (2008–present).

**VITATOPS trial investigators**

*Australia* G J Hankey†, K Loh (Royal Perth Hospital, Perth, WA, number of patients 484); D Crimmins* (Central Coast Neuroscience Research, Gosford, NSW, 102); T Davis*, M England, V Rakic (Fremantle Hospital, Perth, WA, 63); D W Schultz* (Flinders Medical Centre and Griffith Rehabilitation Hospital, Adelaide, SA, 53); J Frayne* (Alfred Hospital, Melbourne, VIC, 42); C Bladin* (Box Hill Hospital, Melbourne, VIC, 42); J Kokkinos* (Bankstown Hospital, Sydney, NSW, 36); D Dunbabin* (Royal Hobart Hospital, Hobart, TAS, 36); J Harper*, P Rees, D Warden (Joondalup Health Campus, Perth, WA, 29); C Levi*, M Parsons, M Russell, N Spratt (John Hunter Hospital, Newcastle, NSW, 26); P Clayton, P Nayagam*, J Sharp (Beleura Private and Frankston Hospitals, Mornington, VIC, 25); K Grainger* (Sir Charles Gairdner Hospital, Perth, WA, 16); C de Wytt* (Greenslopes Private Hospital, Brisbane, QLD, 12); A McDougall* (Liverpool Hospital, Sydney, NSW, 4); G A Donnan* (National Stroke Research Institute-Austin Health, Melbourne, VIC, 4);

R Grimley*, E Neynens* (deceased) (Nambour General Hospital, Nambour, QLD, 2); *Austria* B Reinhart, S Ropele, R Schmidt†, E Stögerer (Medical University of Graz, Graz, 178); *Belgium* P Dedeken, C Schelstraete, G Vanhooren†, A Veyt (AZ Sint-Jan AV, Bruges, 67); *Brazil* C Andre, G R de Freitas†, S E Gomes (Universidade Federal do Rio de Janeiro/Universidade Federal Fluminense/Instituto D’Or de Pesquisa e Ensino, Rio de Janeiro, 71); *China* V C T Mok, A Wong, L K S Wong† (Prince of Wales Hospital, Hong Kong Special Administrative Region, 122); R T F Cheung†, L S W Li (Queen Mary Hospital, Hong Kong Special Administrative Region, 22); *India* P Pais†, D Xavier† (St John’s Medical College and Research Institute, Bangalore, coordinated 23 centres); S Joshi*, S Parthasaradhi (Mahavir Hospital and Research Centre, Hyderabad, Andhra Pradesh, 204); A K Roy*, R V Varghese (St John’s

Medical College Hospital, Bangalore, Karnataka, 123); K Kochar*, R B Panwar (Sardar Patel Medical College and Associated Group of Hospitals, Bikaner, Rajasthan, 117); N Chidambaram*, U Rajasekaharan; (Rajah Muthiah Medical College and Hospital, Annamalai Nagar, Tamilnadu, 109), S Bala, J D Pandian, Y Singh* (Christian Medical College and Hospital, Tamil Nadu, 99); U Karadan, A Salam* (Baby Memorial Hospital, Kerala, 92); S Shivkumar, A Sundararajan* (Neuro Centre, Trichy, Tiruchirapalli, Tamil Nadu, 82); R Joshi, S P Kalantri* (Mahatma

Gandhi Institute of Medical Sciences, Sevagram, Maharashtra, 78); H Singh* (Sadbhavna Medical and Heart Institute, Patiala, Punjab, 70); J M K Murthy*, A Rath (Care Hospital, Hyderabad, Andhra Pradesh, 65); N T R Balasubramanian, A Kalanidhi* (Railway Hospital Perambur, Chennai, Tamil Nadu, 52); K Babu* (Care Hospital, Visakhapatnam, Andhra Pradesh, 46); A Bharani*, P Choudhary, M Jain (Mahatma Gandhi Memorial Medical College and Maharaja Yashwantrao Hospital, Indore, Madhya Pradesh, 39); A Agarwal, M Singh* (Chhatrapati Shahuji Maharaj

Medical University, Lucknow, Uttar Pradesh, 38); R R Agarwal, R Gupta* (Monilek Hospital and Research Centre, Jaipur, Rajasthan, 30); S Kothari*, S Mijar (Poona Hospital, Pune, Maharashtra, 30); S Bandhishti, R S Wadia* (Ruby Hall Clinic, Pune, Maharashtra, 27); S K Paul, S Sekhar Nandi* (Centauri, The Albert Road Clinic, Kolkata, 26); M M Mehndiratta* (GB Pant Hospital, Indraprastha HO, Delhi, 25);

U Tukaram* (Mediciti Hospital, Hyderabad, Andhra Pradesh, 24); K Mittal, A Rohatgi* (Sir Ganga Ram Hospital, New Delhi, Delhi, 21); S Kumar*, K P Vinayan (Amrita Institute of Medical Sciences, Cochin, Kerala, 19); R S Muralidharan* (KS Hospital, Bangalore, Karnataka, 2); *Italy* M G Celani, L Favorito, T Mazzoli, S Ricci†, E Righetti (Perugia Stroke Service, Perugia, 73); M Blundo, A Carnemolla, G D’Asta, A Giordano, F Iemolo* (Ospedale R Guzzardi, Vittoria, 32); M G Celani,

L Favorito, T Mazzoli, S Ricci†, E Righetti (Citta’ della Pieve Stroke Service, Citta’ della Pieve, 23); P Gresele*, F Guercini (University of Perugia, Perugia, 20); R Caporalini, L De Dominicis*, M Giovagnetti, G Giuliani*, S Paoletti, E Pucci (Ospedale di Macerata, Macerata, 18); A Cavallini*, A Persico (IRCCS C Mondino, Pavia, 16); F Casoni, A Costa*, M Magoni*, R Spezi, R Tortorella, E Venturelli, V Vergani (Spedali Civili di Brescia, Bresica, 9); S Caprioli, M Provisione, D Zanotta* (Ospedale di Circolo, Busto Arsizio, 5); *Malaysia* J M Abdullah*, T Damitri, B Idris*, S Sayuthi (Hospital Universiti Sains of Malaysia, Kubang Kerian, 68); J J H Hong, C T Tan, K S Tan† (University of Malaya Medical Centre, Kuala Lumpur,

Selangor, 13); *Moldova* G Dutca, V Grigor, S Groppa†, D Manea (City Emergency Hospital, Chisinau, 114); *Netherlands* S Achterberg, A Algra†, P H A Halkes, L J Kappelle* (University Medical Center Utrecht, Utrecht, 61); A M Boon, J C Doelman, R Sips*, F Visscher (Oosterscheldeziekenhuis, Goes, 37); V I H Kwa*, O A Ternede, J J van der Sande (Slotervaartziekenhuis, Amsterdam, 14); *New Zealand*

T Frendin, J Gommans† (Hawke’s Bay Hospital, Hastings, 101); N E Anderson*, P Bennett, A Charleston, D Spriggs (Auckland City Hospital, Auckland, 62); J Singh* (North Shore Hospital, North Shore, 12); J Bourke*, R Bucknell (Palmerston North Hospital, Palmerston North, 6); H McNaughton* (Wellington Hospital, Wellington, 3); *Pakistan* A Anwar, H Murtaza, W Uddin† (Pakistan Ordinance Factories Hospital, Wah Cantt, Wah, 140); J Ismail* (Dow University of Health Sciences Civil Hospital,

Karachi, 89); N U Khan* (KRL University, Islamabad, 2); *Philippines* J C Navarro† (Jose R Reyes Memorial Medical Center, Manila, 411); V G Amor, M T Canete*, C Lim, E B Ravelo, M Siguenza, M O Villahermosa (Chong Hua Hospital, Cebu City, 137); M T Canete*, M J T Cardino, R Cenabre, M Gara, Z Salas (Cebu Velez General Hospital/Visayas Community Medical Center, Cebu City, 126); A Batac, M T Canete*, L Conde, P Dumdum, F S Garcia, S Libarnes, N Matig-a, N Olanda (Cebu Doctor’s Hospital, Cebu City, 113); R Arcenas, M T Canete*, A Loraña (Vicente Sotto Memorial Medical Center, Cebu City, 104); A Surdilla* (Cagayan de Oro Medical Center, Cebu City, 32); M L Araullo, J Lokin* (University of Santo Tomas Hospital, Manila, 13); G Maylem* (Cagayan Valley Medical Center, Tuguegarao, 1); *Portugal* E Marques, M Veloso* (Hospital Distrital Oliveira de Azeméis, Oliveira de

Azeméis, 61); M Correia†, G Lopes (Hospital Geral de Santo António, Porto, 35); P Canhão, J M Ferro†, T P Melo (Hospital de Santa Maria, Porto, 27); A Dias, A P Sousa* (Hospital Visconde de Salreu, Estarreja, 13); *Georgia* A Tsiskaridze†, T Vashadze (Sarajishvili Institute of Neurology, Tbilisi, 118); *Serbia* I Divjak† (University of Novi Sad [Neurology], Novi Sad, 67); I Divjak†, V Papic (University of Novi Sad [Neurosurgery], Novi Sad, 40); *Singapore* H M Chang, C P L H Chen†, D A De Silva, E K Tan*, M C Wong (Singapore General Hospital, 875); *Sri Lanka* U K Ranawaka†, J C Wijesekera (National Hospital of Sri Lanka, Colombo, 274); H A de Silva*, U K Ranawaka†, C N Wijekoon (University of Kelaniya, Columbo, 87); *UK* J Dawson, P Higgins, K R Lees†, L MacDonald, K McArthur, Y McIlvenna, T Quinn, M Walters (Western Infirmary/ University of Glasgow, Glasgow, 432); R Curless*, J Dickson, J Murdy, A Scott (North Tyneside District Hospital, North Shields, Tyne And Wear, 195); S Cameron, K Darnley, M Dennis*, D Lyle (Western General Hospital, Edinburgh, 161); A Hunter, M Watt*, I Wiggam (Royal Victoria

Hospital, Edinburgh, 118); J Murdy, H Rodgers* (Royal Victoria Infirmary, Newcastle, 97); F Dick, M Macleod, A McKenzie* (Stirling Royal Infirmary, Stirling, 71); P Jones*, S Jones (Bronglais General Hospital, Aberystwyth, 62); L Caudwell, M Hussain* (Musgrove Park Hospital, Taunton, 62); M K Albazzaz*, K Elliott, B Hardware (Barnsley District Hospital, Barnsley, 60); E Bacabac, H Martin, A Sharma*, V Sutton (University Hospital Aintree, Liverpool, 58); H Baht, L Cowie, G Gunathilagan, D R Hargrove, D G Smithard* (William Harvey Hospital, Ashford, Kent, 58); M Adrian, P Bath*, F Hammonds (Nottingham University Hospitals,

Nottingham, 51); H Maguire, C Roff e*, J Rushton (University Hospital of North Staffordshire, Stoke-on-Trent, 43); M Datta-chaudhuri, K Diyazee, S Krishnamoorthy* (Stepping Hill Hospital, Stockport, 42); K McNulty, J Okwera* (Rotherham General Hospital, Rotherham, 39); C Hilaire, D Kelly* (Torbay Hospital, Torbay, 38); L Barron, M James*, N Wedge (Royal Devon and Exeter Hospital, Exeter, 37); M Bruce, M Macleod* (Aberdeen Royal Infi rmary, Aberdeen, 29); M Barber*, D Esson (Monklands Hospital, North Lanarkshire, 19); D Ames, J Chataway* (St Mary’s Paddington Hospital, London, 17); S Bulley, K Jenkins,

K Rashed* (Yeovil Hospital, Yeovil, 15); B E A Dafalla*, T C Venugopalan (St Luke’s Hospital, Crosland Moor, Huddersfield, 14); M Ball, S Punnoose* (Chesterfield Hospital, 13); F Justin, L Sekaran*, S Sethuraman (Luton and Dunstable NHSFT Hospital, Luton, 13); H Goddard, J Howard, J McIlmoyle* (Blackpool Victoria Hospital, Blackpool, 11); C Diver-Hall, M McCarron*, M P McNicholl (Altnagelvin Hospital, Londonderry, 8); B Clamp, J Hunter, A Oke*, K Weaver (Cannock Chase Hospital, Cannock, 7); P Fraser, C McAlpine* (Stobhill Hospital,

Glasgow, 6); J Chambers*, H Dymond, G Saunders (Weston General Hospital, Weston-super-Mare, 6); P Langhorne*, D Stott, F Wright (Glasgow Royal Infirmary, Glasgow, 5); K Adie, R Bland, G Courtauld, F Harrington*, A James, A Mate, C Schofield, C Wroath (Royal Cornwall Hospital, Cornwall, 5); S Duberley, S Punekar* (Royal Preston Hospital, Preston, 5); K Niranjan* (Barking Hospital, Redbridge, 1); D Sandler* (Birmingham Heartlands Hospital, Birmingham, 1); *USA* P Krishna, M Moussouttas* (JFK Hospital, Atlantis, FL, 21); M A Notestine, A Slivka†

(Ohio State University Medical Center, Columbus, OH, 15); D Vallini* (South Carolina VA Hospital, Columbia, SC, 12); T Hwang*, M Saverance (University of South Carolina, Columbia, SC, 7); K Booth*, D Murphy (Abington Memorial Hospital, Abington, PA, 4).

*Principal investigator. †National coordinator.
